# Isolation and exploitation of minority: Game theoretical analysis

**DOI:** 10.1371/journal.pone.0205241

**Published:** 2018-10-05

**Authors:** Dongryul Lee, Pilwon Kim

**Affiliations:** 1 Department of Economics, Sungshin University, Seoul 136-742, Korea; 2 Department of Mathematical Sciences, School of Natural Science, UNIST (Ulsan National Institute of Science and Technology), Ulsan 689-798, Korea; Universidad Veracruzana, MEXICO

## Abstract

We investigate various group-size distributions occurring in a situation where each group’s resource is exposed to appropriation by other groups. The amount of appropriation depends on the size difference between groups. Our work focuses on the cases where the entire community isolates a small group or even an individual to maximize its gain. While people’s basic motivation to form a group can be understood based on the group-size effect on multiplying a collective asset, sensitive factors that induce a asymmetric group distribution are the group efficiency and the ratio of secured assets to assets pending in a competition. We show that social rejection to a minor group may occur when the group efficiency is relatively low and their asset is severely exposed to possible appropriation.

## Introduction

Minority denotes ostracized individuals or groups that are inferior to the rest of the population in terms of their power, numeric size, group features, group conditions and treatment [1, 2]. Discrimination and hostility against certain groups of people has been common practice, observed across different societies [3–5].

There are two types of Economic models of discrimination: competitive models and collective models [4]. While most of economic analysis is based on competitive model and has focused on individual behavior inducing discrimination [6–8], there has been relatively few work on collective models dealing with collective group interactions.

In the context of the game theory, social exclusion and segregation has been mainly dealt as an effective control of defectors [9–12]. While the most approaches have treated peer rejection as an effective mean of punishment for free-riders, there have been not many studies in the context of group formation process. In [13], the author used a stochastic group-forming model to describe passive rejection and showed that people’s thrust of belongingness and homophily can create an accidental outcast.

In this paper, we investigate isolation of minorities and active social rejection to an individual occurring in group formation in game theoretical framework. That is, we first define the payoffs for each individuals, given every perceivable group (coalition) structures, and then check if each group structure is stable or not, as in the conventional game-theoretic analysis on group formation [14–19]. We analyse effects of the relative group size on conflicts and appropriations between groups. The emerging literature on political economics on tradeoff between production and appropriation has shown that conflict in economic interactions imposes profound implications for the distribution of resources [20–23]. Especially in [24], the influence of distributional conflict in a “winner-take-all” contest on alliance formation has been studied.

We study how group of various size forms if each group’s resource is partially exposed to appropriation. In the presence of conflicts, people need to decide their group size compromising between performance, appropriation, and security. One of our main concern is to develop a minimal model that explains the ubiquity of the minority isolation. As such, we focus on how the relative size of groups affects on group productions and conflicts, while assuming that all the members in groups equally share their product and loss.

## Model

### Benefit of forming groups

One of the main benefits of forming a group is to multiply a collective output by making better use of members’ skills and resources through cooperation. We assume the output of a group of *n* persons is

$$\begin{array}{c} c\;n^{\alpha } \end{array}$$

where *c* > 0 is a proportional constant. We call an exponent *α* ≥ 0 *the group efficiency*, which represents the degree of effectiveness of the group size in output. For *α* = 1, the performance of the group is simply proportional to its size. As such, the group size essentially has the null effect on the group production. If *α* > 1, *n*^*α*^ exhibits increasing returns to scale and the size of each group has a greater effect on its share than for *α* = 1. This gives an motivation to organize as a large group as possible, since the members mean share increase accordingly. On the contrary, the managing cost for large groups can grow faster than the group size, for example, due to bureaucratic inefficiency. If 0 ≤ *α* < 1, forming a group is not an attractive choice to people as their mean share reduces with the group size, unless there is further motivation. From here on, we assume that the proportional coefficient *c* is one for convenience. We additionally assume that there is a group income tax paid for the central maintenance and service. A group of *n* persons pays the tax

$$\begin{array}{c} r_{T}\;n^{\alpha } \end{array}$$

which is proportional to the group output *n*^*α*^ with the tax rate *r*_T_, 0 ≤ *r*_T_ ≤ 1. The tax in the model represents a fixed-rate expenditure proportional to an absolute group size.

### Conflict-induced wealth redistribution

Wealth redistribution between groups, which is induced by group-group conflicts, contrasts with the tax and depends on a relative group size. Suppose *N* people forms *K* exclusive groups, *n*_1_, *n*_2_, ⋯, *n*_*K*_ with ∑_*k* = 1_ *n*_*k*_ = *N* ≥ 3. We consider a situation where groups directly confronts one another and a part of their group products, $r_{C}\;n_{i}^{\alpha },0\leq r_{C}\leq 1$ are exposed to mutual appropriation. That is, here *r*_C_ represents the ratio of the assets subject to appropriation, reflecting intensity of conflicts.

We assume that, in conflicts, a group’s gain(loss) against another group depends on their size difference. To be more specific, suppose that group *i* and group *j* confront each other and group *i* is larger than group *j*. We assume that group *i* deprives group *j* of its exposed asset $r_{C}\;n_{j}^{\alpha }$ in proportion of their relative size difference (*n*_*i*_ − *n*_*j*_)/*N*. Hence the amount of asset that group *i* takes away from group *j* is

$$\begin{array}{c} r_{C}\;\frac{n_{i}-n_{j}}{N}n_{j}^{\alpha }. \end{array}$$

The total asset of group *i* is now obtained by summing up the above values: the original product $n_{i}^{\alpha }$ minus the income tax $r_{T}\;n_{i}^{\alpha }$, the gain from the small groups $r_{C}\left(n_{i}-n_{k}\right)n_{k}^{\alpha }/N,n_{i}>n_{k}$, and the loss to the larger groups $r_{C}\left(n_{k}-n_{i}\right)n_{i}^{\alpha }/N,n_{k}>n_{i}$. This can be formulated as

$$\begin{array}{ccc} g_{i} & = & n_{i}^{\alpha }-r_{T}\;n_{i}^{\alpha }+\frac{r_{C}}{N}\sum_{k=1,\;n_{i}>n_{k}}^{K}\left(n_{i}-n_{k}\right)n_{k}^{\alpha }-\frac{r_{C}}{N}\sum_{k=1,\;n_{i}<n_{k}}^{K}\left(n_{k}-n_{i}\right)n_{i}^{\alpha } \end{array}$$ (1)

$$\begin{array}{ccc} & = & \left(1-r_{T}\right)n_{i}^{\alpha }+\frac{r_{C}}{N}\sum_{k=1}^{K}\left(n_{i}-n_{k}\right)\min\left\{n_{i}^{\alpha },n_{k}^{\alpha }\right\}. \end{array}$$ (2)

Note that the amounts of the tax and the appropriation do not directly affect each other. While the amount of the tax is determined from the absolute size of the group, what determines the appropriation is the relative size of the group.

Now we define the individual payoff of a member in group *i* as *π*_*i*_ = *g*_*i*_/*n*_*i*_, assuming that the wealth in the group is equally shared. That is,

$$\begin{array}{ccc} \pi_{i} & = & \frac{g_{i}}{n_{i}} \end{array}$$ (3)

$$\begin{array}{ccc} & = & \left(1-r_{T}\right)n_{i}^{\alpha -1}+\frac{r_{C}}{N}\sum_{k=1}^{K}\left(n_{i}-n_{k}\right)\min\{n_{i}^{\alpha -1},\frac{n_{k}^{\alpha }}{n_{i}}\} \end{array}$$ (4)

Note that introducing group conflicts and appropriation may twist the group size effect. For example, we confirmed above that no group spontaneously forms with the small group-size effect, 0 ≤ *α* < 1. This is no more true with conflicts existing, since their smallness makes groups more vulnerable to appropriation. Even when forming a group does not promote efficient productions in output, people can make an alliance to protect their assets.

If *r*_C_ is low, conflicts between groups are not severe, and a relatively large part of the group production can be secured for each group. On the contrary, a high value of *r*_C_ indicates that the society is driven by intensive conflicts and the large portion of the assets is subject to appropriation by other groups. Especially when *r*_C_ > 1 − *r*_T_, the society is driven by intensive conflicts and the situation becomes very harsh to minor groups. Most of their properties is at risk of being taken by larger groups. What is even worse is that they have to pay out the fixed-rate tax, regardless of how much they lost in the conflicts. This implies that some small groups unavoidably end up with a negative payoff.

### Group dynamics

People change or create their groups to increase their individual payoff. We assume that an individual can deviate from one group and get in another, only with the consent of the people in the group that he/she wants to join. This implies that transfer can occur only when it leads to increase in payoffs of both the new comer and existing members. In addition, an individual can escape from a group and create a singleton, if staying alone is more beneficial than remaining in the group. Group dynamics is a serial combination of such individual movements. We are interested in searching Nash equilibriums of group distributions where no individual has incentive to change their groups any more.

## Result

The group distribution at Nash equilibrium varies widely with respect to the group efficiency *α* and the conflict intensity *r*_C_. We divide the cases below into low/high conflict society with low/neutral/high group efficiency.

### Society with neutral group efficiency (*α* = 1)

In case that the group product linearly increases with the group size, all possible group distributions are equally attractive and people are indifferent in changing their groups. However, the presence of group conflicts and central taxation makes the situation more complicated. People further have to consider possible appropriation between them when forming groups. As a result, there are two groups left in the end, of which size ratio may vary.

**Theorem 1** *Suppose α* = 1. *Let n*^0^ *be the size of the largest group in the initial group distribution. Then the group distribution converges to a two-group formation*, (n*, *N* − n*)*, where* $n^{*}=\max\left\{n^{0},\left\lceil \sqrt{N^{2}/2+1/4}-1/2\right\rceil \right\}$.

Here ⌈*x*⌉ denotes the smallest integer greater than or equal to *x*. According to Theorem 1, distributions with more than three groups are unstable and bound to merge into a two-group formation. The size of the larger group is $\sqrt{N^{2}/2+1/4}-1/2)\approx 0.7N$ or greater. Once there appears a group larger than 70% of the population, the members have no incentive to accept a new comer. That is, unless the initial group formation includes a larger group from the beginning, the distribution ends up with the 70:30 formation. It is notable that the asymmetric size ratio depends only on the initial condition *n*^0^, not on either *r*_T_ or *r*_C_, although the result cannot be achieved without the presence of group conflicts and central taxation,

### Society with high group efficiency (*α* > 1)

The desire to unify grows with the group efficiency. For an intermediate value of *α* > 1, people tend to create a large group to maximize the group output. This motivation competes with a tendency to leave out small groups outside so that they can appropriate the small group’s production. However, with high values of *α*, the benefit of unifying groups overwhelms the potential appropriation.

**Theorem 2** *For sufficiently large α* > 1, *the only Nash equilibrium is single grand group distribution*.

### Low conflict society with low group efficiency (*α* < 1, *r*_C_ ≤ 1 − *r*_T_)

When the group efficiency is less than 1, *α* < 1, the situation becomes more complex. Although people basically want to stand alone due to low group efficiency, still in the presence of conflicts, they further have to seek security against appropriation. This motivation leads to two completely different situations, depending the intensity of conflict *r*_C_.

If conflicts between group are rather weak, multiple groups commonly appear. As *α* decreases below 1, two-group formations in Theorem 1 are bound to break, making more various group formations possible. For example, if *N* = 100, *r*_T_ = *r*_C_ = 4/9 and *α* = 4/5, the group distributions such as (83, 17), (74, 20, 6), (63, 29, 7, 1) are all possible Nash equilibriums. Still, in this regime of mild competition, *r*_C_ ≤ 1 − *r*_T_, an extremely low group efficiency makes people scatter, giving up grouping:

**Theorem 3** *Suppose* *r*_C_ ≤ 1 − *r*_T_. *For sufficiently small α* < 1, *the groups break into all singletons*.

### Conflict-driven society with low group efficiency (*α* < 1, *r*_C_ > 1 − *r*_T_)

When *α* is small, the group formation depends on *r*_C_ and drastically changes across the value *r*_C_ = 1 − *r*_T_. If *r*_C_ > 1 − *r*_T_, appropriation of other groups becomes a dominant part of the group members’ gain. In the appropriation, since the group efficiency is low, more significant factor is how much bigger their group is than their opponents. They concern the size difference, rather than opponent’s absolute size and the corresponding output. That is, the situation becomes close to a pure power game. The group distribution ends up with an asymmetric formation that consists of one large group and several minor groups. For example, (80, 2, 2, 1, …1) is one typical equilibrium.

As *α* decreases, the size gap gets widened. Especially in the limit of *α* → 0, with a little strong condition as $r_{C}>\frac{N}{N-2}\left(1-r_{T}\right)$, all small groups are eventually merging to the largest group, leaving one unfortunate individual as an outcast.

**Theorem 4** *Suppose* *r*_C_ > 1 − *r*_T_. *For sufficiently small α* < 1, *there is at most one group of which size is greater than 1. Moreover, suppose the initial distribution is not the single grand group and suppose a slightly stronger condition* $r_{T}>1-\frac{N-2}{N}r_{C}$ *holds. Then the only Nash equilibrium is (N* − 1, 1).

This indicates occurrence of the social rejection, like (99, 1). The absolute portion of the profits that the members of the larger group share is from exploitation of the isolated individual. Theorem 4 implies that people form a large group and isolate an outcast when they find no intrinsic benefits in forming a group.

When a large portion of their asset is exposed to severe competition, people start to use the exclusion and isolation strategy. They aggregate not because they can produce more, but because they can deprive more. The model indeed reveals a subtle point regarding social exclusion: in a predatory economy, a welfare gap between being a part of a larger group and being an isolated individual becomes extremely large. One is made to cooperate to exclude someone else, in fear of others victimizing him or her first.

## Discussion

In this study, we investigate the group formation in the situation where the assets of the group are subject to appropriation in conflicts. It is assumed that the absolute size of each group determines the first-hand group production and the relative size determines the second-hand redistribution. We are especially interested in finding conditions for extremely asymmetric group distributions, and it turns out that isolation of minority occurs if the group efficiency is low and the portion of the assets at risk of loss is high in conflicts. It is an irony that people form an exclusive group and create a minority, when there is no intrinsic benefits of forming a group like promoting productivity through internal collaboration. They do such things because rejection and appropriation is a better way to raise their benefits. This explains why social ostracism more frequently occurs in educational, political or cultural communities. Conflicts in the communities based on non-financial relations often take a form of a pure power game. The effect of bullying/ridiculing depends on superiority in numbers and tends to be multiplied with the participants.

Social exclusion occurs when society has no secure measure to protect the groups’ assets from competitions and leaves no other way to raise productivity, except appropriation. Within the context of the model, the social planner can reduce occurrence of the minority isolation through adjusting the group efficiency *α*, the central tax *r*_T_, and the intensity of conflicts *r*_C_. First, she needs to promote the intrinsic benefit of forming a group. Getting together should be good by itself, not by comparing with others. If possible, the central managing cost *r*_T_ should be cut down. Also, conflicts between groups needs to be properly controlled so that a part of group assets can be protected from unlimited appropriation. As long as kept at a suitable level, competitions and conflicts between groups may give a diversity in group distribution.

## Supporting information

S1 Appendix Proofs of Theorems. (PDF)
