# Supplementary material for: Isolation and exploitation of minority: Game theoretical analysis
Source: PLoS One. 2018 Oct 5;13(10):e0205241. doi: 10.1371/journal.pone.0205241 (PMC6173431; doi:10.1371/journal.pone.0205241)
Supplement: S1 Appendix — (PDF) [file pone.0205241.s001.pdf]

Supplement for the paper

Isolation and Exploitation of Minority: Game Theoretical Analysis

by: Pilwon Kim and Dongryul Lee

Theorem 1. *Suppose* $\alpha=1$. *Let* n^0 *be the size of the largest group in the initial group distribution. Then the group distribution converges to a two-group formation,* $(n^*, N-n^*)$, *where* $n^* = \max(n^0, \lceil \sqrt{N^2/2+1/4}-1/2 \rceil)$.

Lemma 1. *Suppose* $\alpha=1$. *If* $n_i \le n_j$ *then* $\pi_i \le \pi_j$. *This implies that a member of a larger group never transfers to a smaller group.*

Proof.
Observe

$$\pi_i = (1-r_T) + \frac{r_C}{N}\sum_{\substack{k \\ n_i > n_k}} (n_i - n_k)\frac{n_k}{n_i} + \frac{r_C}{N}\sum_{\substack{k \\ n_i < n_k}} (n_i - n_k)$$

$$= (1-r_T) + \frac{r_C}{N}\sum_{k=1}^{K}(n_i - n_k)\min\left\{1, \frac{n_k}{n_i}\right\}$$

Then

$$\pi_j - \pi_i = \frac{r_C}{N}\sum_{k=1}^{K} f_{i,j}^k \tag{1}$$

where

$$f_{i,j}^k = (n_j - n_k)\min\left\{1, \frac{n_k}{n_j}\right\} - (n_i - n_k)\min\left\{1, \frac{n_k}{n_i}\right\}$$

We decompose the summation in the right hand side of (1) as

$$\sum_{k=1}^{K} f_{i,j}^k = \sum_{\substack{k \\ n_k \le n_i}} f_{i,j}^k + \sum_{\substack{k \\ n_i \le n_k \le n_j}} f_{i,j}^k + \sum_{\substack{k \\ n_j \le n_k}} f_{i,j}^k .$$

Three parts are all positive since

$$\sum_{\substack{k \\ n_k \le n_i}} f_{i,j}^k = \sum_{\substack{k \\ n_k \le n_i}} \left((n_j - n_k)\frac{n_k}{n_j} - (n_i - n_k)\frac{n_k}{n_i} \right)$$

$$= \sum_{\substack{k \\ n_k \le n_i}} \frac{n_j - n_i}{n_i n_j}$$

$$\ge 0$$

$$\sum_{\substack{k \\ n_i \le n_k \le n_j}} f_{i,j}^k = \sum_{\substack{k \\ n_i \le n_k \le n_j}} \left((n_j - n_k)\frac{n_k}{n_j} - (n_i - n_k) \right)$$

$$\ge 0$$

$$\sum_{\substack{k \\ n_j \le n_k}} f_{i,j}^k = \sum_{\substack{k \\ n_j \le n_k}} \left((n_j - n_k) - (n_i - n_k)\right)$$

$$= \sum_{\substack{k \\ n_j \le n_k}} (n_j - n_i)$$

$$\ge 0$$

■

Lemma 2. Suppose $\alpha = 1$. Any distribution group distribution that consists of more than two groups evolves into a two-group formation.

Proof.

Suppose there are more than two groups at Nash equilibrium, that is, $K \ge 3$. Let group 1 and 2 be two smallest groups with $1 \le n_1 \le n_2$. Note $n_1, n_2 < N/2$. It suffices to show that there is always incentive for a member of group 1 to join the group 2 and such transfer is granted by the group 2. If so, every group distribution that consists of more than two groups are not stable.

Let $\widehat{\pi_2}$ denote the updated payoff of the group 2 in case a member of the group 1 joins. Since $\pi_1 \le \pi_2$ from Lemma 1, it is enough to show $\pi_2 < \widehat{\pi_2}$

$$\pi_2 = (1 - r_t) n_2 + \frac{r_c}{N} \frac{n_2 - n_1}{n_2} n_1 - \frac{r_c}{N} \sum_{k=3}^{K} (n_k - n_2)$$

$$= (1 - r_t) n_2 + \frac{r_c}{N} + \frac{r_c}{N} \frac{n_1^2}{n_2} - \frac{r_c}{N} (N - n_1 - n_2) + \frac{r_c}{N} (K - 2) n_2$$

$$\hat{\pi_2} = (1 - r_T)(n_2 + 1) + \frac{r_C}{N} \frac{n_2 - n_1 + 2}{n_2 + 1} (n_1 - 1) - \frac{r_C}{N} \sum_{k=3}^{K} (n_k - n_2 - 1)$$

$$= (1 - r_T)(n_2 + 1) + \frac{r_C}{N} + \frac{r_C}{N} \frac{(n_1 - 1)^2}{n_2 + 1} - \frac{r_C}{N} (N - n_1 - n_2)$$

$$+ \frac{r_C}{N} (K - 2) n_2 + \frac{r_C}{N} (K - 2)$$

$$\widehat{\pi_2} - \pi_2 = 1 - r_T + \frac{r_C}{N} (K - 2) + \frac{r_C}{N} \left(\frac{(n_1 - 1)^2}{n_2 + 1} - \frac{n_1^2}{n_2} \right)$$

$$= 1 - r_T + \frac{r_C}{N} (K - 2) + \frac{r_C}{N} \frac{n_1^2 + (2n_1 - 1) n_2}{n_2 (n_2 + 1)}$$

$$> 0.$$

This implies that as long as $K \ge 3$, the smallest group is always to be absorbed into other

groups and therefore there are only two group left in the end. ■

Lemma 3. Suppose $\alpha = 1$. If there exists a group greater than $n \geq N_c$ where $N_c = \sqrt{N^2/2 + 1/4} - 1/2$, then there is no incentive for its members to accept a new member. That is, no group of which size is greater than n^* forms unless there is one from the beginning.

Proof.

Suppose that the group j is the largest group and its size n_j is greater than n^*. Let π_j be the individual payoff of the group j and let $\hat{\pi}_j$ denote the updated payoff of the group j in case a member of the group i joins. Observe

$$\pi_j = (1 - r_T) + \frac{r_C}{N}(n_j - n_i)\frac{n_i}{n_j} + \frac{r_C}{N}\sum_{k \neq i}^{K}(n_j - n_k)\frac{n_k}{n_j}$$

and

$$\hat{\pi}_j = (1 - r_T) + \frac{r_C}{N}(n_j - n_i + 2)\frac{n_i - 1}{n_j + 1} + \frac{r_C}{N}\sum_{k \neq i}^{K}(n_j - n_k + 1)\frac{n_k}{n_j + 1}.$$

Then

$$\begin{aligned}
\pi_j - \hat{\pi}_j &= \frac{r_C}{N}\frac{1}{n_j(n_j+1)}\Big((n_j - n_i)n_i(n_j+1) - (n_j - n_i + 2)(n_i - 1)n_j \\
&\qquad + \sum_{k \neq i}^{K}((n_j - n_k)n_k(n_j+1) - (n_j - n_k + 1)n_k n_j) \Big) \\
&= \frac{r_C}{N}\frac{1}{n_j(n_j+1)}\Big(n_j(n_j - 2n_i + 2) - \sum_{k=1}^{K} n_k^2\Big) \\
&\geq \frac{r_C}{N}\frac{1}{n_j(n_j+1)}\big(n_j(n_j - 2(N - n_j) + 2) - (N - n_j)^2\big) \\
&\geq \frac{r_C}{N}\frac{1}{n_j(n_j+1)}(2n_j^2 + 2n_j - N^2).
\end{aligned}$$

Hence, we confirm $\pi_j - \hat{\pi}_j \geq 0$ if $2n_j^2 + 2n_j N - N^2 \geq 0$, or

$$n_j \geq N_c \tag{2}$$

where $N_c \equiv \sqrt{N^2/2 + 1/4} - 1/2$. ■

Proof of Theorem 1.

Lemma 2 implies that multiple groups gradually unify into two groups: Such two group formation never breaks back to multiple groups, only allowing the member transfer between two groups. Let $N_c \equiv \sqrt{N^2/2+1/4}-1/2$.

i) Suppose $n^0 \geq N_c$. From Lemma 1, transfer from this group to smaller group is disadvantageous to the members. Lemma 3 implies that the group never allows transfer-in. Hence the group remains the same while all other groups merge. The corresponding final group distribution is $(n^0, N-n^0)$.

ii) Suppose $n^0 < N_c$. Let (n_1,n_2), $n_1 \leq n_2$ be a two-group distribution which the corresponding initial distribution evolves into. Since merging of groups only occurs through transfer of individual, n_2 cannot exceed N_c from Lemma 3. Denote π_1 and π_2 as the corresponding individual payoffs of two groups, respectively. Also let $\widehat{\pi}_1$ and $\widehat{\pi}_2$ be the individual payoffs of the group distribution (n_1-1, n_2+1). Note that, if $\pi_2 < \widehat{\pi}_2$, then there is a transfer from group 1 to group 2. Observe

$$\pi_2 = 1 - r_T + \frac{r_C}{N}(n_2 - n_1)\frac{n_1}{n_2}$$

and

$$\widehat{\pi}_2 = 1 - r_T + \frac{r_C}{N}(n_2 - n_1 + 2)\frac{n_1 - 1}{n_2 + 1}.$$

Then

$$\begin{aligned}\pi_2 - \widehat{\pi}_2 &= \frac{r_C}{N}(n_2 - n_1)\frac{n_1}{n_2} - \frac{r_C}{N}(n_2 - n_1 + 2)\frac{n_1 - 1}{n_2 + 1} \\ &= \frac{r_C}{N}\frac{1}{n_2(n_2+1)}\left((n_2 - n_1)n_1(n_2+1) - (n_2 - n_1 + 2)(n_1 - 1)n_2\right) \\ &= \frac{r_C}{N}\frac{1}{n_2(n_2+1)}\left(2n_2^2 + 2n_2 - N^2\right)\end{aligned}$$

Hence, $\pi_2 - \widehat{\pi}_2 < 0$ equivalently means $2n_2^2 + 2n_2 - N^2 < 0$, or,

$$n_2 < N_c.$$

which is the opposite of the condition (2) in the proof of Lemma 2. This implies that the transfer from the smaller group to the larger group continues until the size of the larger group reaches N_c. Once it becomes such group of which size is no less than N_c, no more transfer occurs from Lemma 3. Hence $(\lceil N_c \rceil, N - \lceil N_c \rceil)$ is the final group distribution. ■

Theorem 2. *For sufficiently large $\alpha > 1$, the only Nash equilibrium is single grand group distribution.*

Proof.
Observe

$$\pi_i = (1-r_T) n_i^{\alpha-1} + \frac{r_C}{N} \sum_{\substack{k \\ n_k < n_i}} (n_i - n_k) \frac{n_k^\alpha}{n_i} + \frac{r_C}{N} \sum_{\substack{k \\ n_k > n_i}} (n_i - n_k) n_i^{\alpha-1}.$$

$$= (1-r_T) n_i^{\alpha-1} + \frac{r_C}{N} \sum_{k \neq i} (n_i - n_k) \min\left\{ n_i^{\alpha-1}, \frac{n_k^\alpha}{n_i} \right\}$$

Suppose jth group is the largest group. Then

$$\pi_j = (1-r_T) n_j^{\alpha-1} + \frac{r_C}{N} \sum_{k \neq j} (n_j - n_k) \frac{n_k^\alpha}{n_i}. \tag{4}$$

We are going to prove that a member in any other group i has an incentive to move in the group j and the group j members willingly accept it. That is, if $\widetilde{\pi_j}$ denotes the individual gain of the group j members in case there is a transfer of one member from the group i to the group j, we need to show $\widetilde{\pi_j} > \pi_j \geq \pi_i$.

First, observe

$$\pi_j - \pi_i = (1-r_T)\left(n_j^{\alpha-1} - n_i^{\alpha-1}\right) + \frac{r_C}{N}\left((n_j - n_i) \frac{n_i^\alpha}{n_j} \right) \tag{5}$$

$$+ \frac{r_C}{N} \sum_{k \neq i,j} \left((n_j - n_k) \frac{n_k^\alpha}{n_j} - (n_i - n_k) \min\left\{ n_i^{\alpha-1}, \frac{n_k^\alpha}{n_i} \right\} \right).$$

It is clear that the first and the second tern in the right hand side of (5) is nonnegative. The term in the summation satisfies, if $n_i \geq n_k$,

$$(n_j - n_k)\frac{n_k^\alpha}{n_j} - (n_i - n_k) \min\left\{ n_i^{\alpha-1}, \frac{n_k^\alpha}{n_i} \right\} = \frac{n_j - n_i}{n_i n_j} n_k^{\alpha+1}$$

$$\geq 0.$$

If $n_i < n_k$,

$$(n_j - n_k)\frac{n_k^\alpha}{n_j} - (n_i - n_k) \min\left\{ n_i^{\alpha-1}, \frac{n_k^\alpha}{n_i} \right\} = (n_j - n_k)\frac{n_k^\alpha}{n_j} + (n_k - n_i) n_i^{\alpha-1}$$

$$> 0.$$

Hence, we have $\pi_j \geq \pi_i$ either way.

Now we need to show adding more member is beneficial to the members of the largest group j. Let $\widetilde{\pi_j}$ denote the individual gain of the group j members in case there is a transfer of

one member from the group i to the group j. Then

$$\widetilde{\pi_j} = (1-r_T)(n_j+1)^{\alpha-1} + \frac{r_C}{N}(n_j-n_i+2)\frac{(n_i-1)^\alpha}{n_j+1} + \frac{r_C}{N}\sum_{k\neq i,j}(n_j-n_k+1)\frac{n_k^\alpha}{n_j+1}.$$

We compare it to the original benefit:

$$\widetilde{\pi_j} - \pi_j = (1-r_T)\left((n_j+1)^{\alpha-1} - n_j^{\alpha-1}\right) \tag{6}$$

$$+\frac{r_C}{N}\left((n_j-n_i+2)\frac{(n_i-1)^\alpha}{n_j+1} - (n_j-n_i)\frac{n_i^\alpha}{n_j}\right)$$

$$+\frac{r_C}{N}\sum_{k\neq i}^{K}\left((n_j-n_k+1)\frac{n_k^\alpha}{n_j+1} - (n_j-n_k)\frac{n_k^\alpha}{n_j}\right)$$

Note that the equation (6) may read as

$$\widetilde{\pi_j} - \pi_j = a\,(n_j+1)^\alpha + b\,n_j^\alpha + c\,(n_j-1)^\alpha + d\,(n_j-2)^\alpha + \cdots,$$

where $a = \frac{1-r_T}{n_j+1} > 0$. This implies $\widetilde{\pi_j} - \pi_j > 0$ for sufficiently large α. That is, for sufficiently large α, we get $\widetilde{\pi_j} > \pi_j \geq \pi_i$.

■

Theorem 3. *Suppose* $r_C > 1 - r_T$. *For sufficiently large* $\alpha > 1$, *the groups break into smaller all singletons.*

Proof.

$$\pi_i = (1 - r_T) n_i^{\alpha-1} + \frac{r_C}{N} \sum_{\substack{k \\ n_i > n_k}} (n_i - n_k) \frac{n_k^\alpha}{n_i} + \frac{r_C}{N} \sum_{\substack{k \\ n_i \le n_k}} (n_i - n_k) n_i^{\alpha-1}$$

$$= (1 - r_T) n_i^{\alpha-1} + \frac{r_C}{N} \sum_{k=1}^{K} (n_i - n_k) \min\left\{ n_i^{\alpha-1}, \frac{n_k^\alpha}{n_i} \right\}$$

This is a continuous function with respect to $\alpha \ge 0$. Hence, the order of the individual payoff at $\alpha = 0$ is preserved for sufficiently small $\alpha \ge 0$.

If $\alpha = 0$,

$$\pi_i = (1 - r_T) \frac{1}{n_i} + \frac{r_C}{N} \sum_{k=1}^{K} (n_i - n_k) \frac{1}{n_i}$$

$$= \frac{1}{n_i} \left(1 - r_T + \frac{r_C}{N} (K n_i - N) \right)$$

$$= \frac{1}{n_i} \left(1 - r_T - r_C + \frac{K}{N} n_i r_C \right)$$

$$= \frac{1}{n_i} (1 - r_T - r_C) + \frac{K}{N} r_C.$$

Note the individual payoff of a group only depends on the size of the corresponding group size, $(1 - r_T - r_C)/n_i$. Hence, if $1 - r_T - r_C > 0$, all members better to deviate and form a singleton, since such deviation raises both $(1 - r_T - r_C)/n_i$ and the value of K simultaneously.

■

Theorem 4. *Suppose* $r_C > 1 - r_T$. *For sufficiently small* $\alpha \geq 0$, *there is at most one group of which size is greater than 1. Moreover, suppose the initial condition is not the single grand group and suppose a slightly stronger condition* $r_C > \frac{N}{N-2}(1-r_T)$ *holds, Then the only Nash equilibrium is* $(N-1,1)$.

Proof.
As seen in the proof of Theorem 3, if $\alpha = 0$,

$$\pi_i = \frac{1}{n_i}(1 - r_T - r_C) + \frac{K}{N} r_C$$

Since $1 - r_T - r_C < 0$, as long as the number of groups K does not decrease, the transfer of a member to a larger group is beneficial to both the member and the accepting group. Hence, all groups lose their members to one large group until they shrink to a singleton.

Now further suppose $r_C > (1 - r_T)\frac{N}{N-2}$. Let π and $\tilde{\pi}$ be the individual payoff of a singleton and that of two singletons after their unification, respectively. One can confirm

$$\pi = 1 - r_T - r_C + \frac{K}{N} r_C \quad \text{and} \quad \tilde{\pi} = \frac{1 - r_T - r_C}{2} + \frac{K-1}{N} r_C .$$

Then

$$\begin{aligned}\tilde{\pi} - \pi &= \frac{r_T + r_C - 1}{2} - \frac{1}{N} r_C \\ &= \frac{1}{2}\left(r_T + \frac{N-2}{N} r_C - 1\right) \\ &> 0.\end{aligned}$$

Hence any two singleton have incentive to merge, leaving no more than one singleton in the end. Now we compare two group distributions $(N-1,1)$ and (N).
The individual payoff of the group of $N-1$ persons in the distribution $(N-1,1)$ is $(1 - r_T - r_C)/(N-1) + 2r_C/N$, while that of the grand group of N persons is $(1 - r_T)/N$. The difference is

$$\begin{aligned}&\frac{1}{N-1}(1 - r_T - r_C) + \frac{2}{N} r_C - \frac{1}{N}(1 - r_T) \\ &= \frac{1}{N(N-1)}\left((N-2) r_C - (1 - r_T)\right) \\ &\geq \frac{1}{N(N-1)}(r_T + r_C - 1) \\ &> 0.\end{aligned}$$

This implies that the group of $N-1$ persons do not absorb the singleton. Hence $(N-1,1)$is the only reachable equilibrium. ■
